# Drug‐Induced Liver Injury Caused by Metamizole: Identification of a Characteristic Injury Pattern

**DOI:** 10.1111/liv.70012

**Published:** 2025-02-06

**Authors:** Sabine Weber, Franziska Erhardt, Julian Allgeier, Didem Saka, Nirali Donga, Jens Neumann, Christian M. Lange, Alexander L. Gerbes

**Affiliations:** ^1^ Department of Medicine II LMU Klinikum Munich Germany; ^2^ Institute of Pathology, Medical Faculty LMU Munich Germany

**Keywords:** drug adverse reactions, drug toxicity, hepatotoxicity, liver injury

## Abstract

**Background and Aims:**

Drug‐induced liver injury (DILI) due to metamizole has gained increasing attention. Causality assessment remains a challenge, especially in patients with co‐medications. We therefore aimed to further characterise metamizole DILI cases.

**Methods:**

The data of patients with metamizole intake from our prospective study on acute liver injury with potential drug‐related causes were analysed. Diagnosis and causality assessment were based on a thorough work‐up and long‐term follow‐up.

**Results:**

DILI was associated with metamizole in 61 of 324 DILI patients (prevalence 18.8%). A highly characteristic clinical pattern was observed in 43 of the 61 patients, characterised by marked elevation of transaminases peaking at the time of DILI recognition and a more pronounced increase of bilirubin within the first 3 days of clinical presentation. Patients fitting this picture had higher rates of jaundice, coagulopathy, and acute liver failure, however outcomes did not differ significantly when compared to non‐metamizole DILI and autoimmune hepatitis (AIH) patients. Overall, fatal adverse outcomes defined by death or liver transplantation were observed in 13.1% of metamizole DILI patients. On multivariate analysis, only aspartate aminotransferase (AST) and INR were independently associated with a fatal adverse outcome. INR, in particular, performed better than Hy's law, bilirubin, transaminases, and the model for end‐stage liver disease (MELD), with a *c*‐statistic of 0.85 (95% CI: 0.70–1.0). At a cut‐off of ≥ 2.1, sensitivity and specificity for a fatal adverse outcome were 75% and 96%, respectively.

**Conclusions:**

Metamizole DILI can present with a characteristic pattern that can help clinicians to identify metamizole as the causative agent. Outcome, however, is not associated with this clinical picture and should rather be predicted by INR at onset.

**Trial Registration:**

ClinicalTrials.gov identifier: NCT 02353455

**Abbreviations**
AIH: autoimmune hepatitis
ALF: acute liver failure
ALI: acute liver injury
ALP: alkaline phosphatase
ALT: alanine aminotransferase
ANA: antinuclear antibodies
AST: aspartate aminotransferase
BMI: body mass index
CI: confidence interval
*c*‐statistic: concordance statistic
DILI: drug‐induced liver injury
IgG: immunoglobulin G
INR: international normalised ratio
LT: liver transplantation
MELD: model for end‐stage liver disease
MRI: magnetic resonance imaging
NPV: negative predictive value
NSAID: non‐steroidal anti‐inflammatory drugs
OR: odds ratio
PPV: positive predictive value
RECAM: revised electronic causality assessment method
ROC: receiver‐operating characteristics
RUCAM: Roussel Uclaf Causality Assessment Method
TBIL: total bilirubin
ULN: upper limit of normal

**Summary**

- Metamizole is an analgesic drug with a known hepatotoxic potential; however identifying metamizole as the cause of liver injury in patients with multiple drug intake can be challenging.
- We detected a typical pattern of laboratory parameters, which can help clinicians to diagnose liver injury caused by metamizole and avoid erroneous association of liver injury with innocent bystander medication.
- Thus, the identification of metamizole as the causative agent can be accelerated, and discontinuation of necessary therapeutic agents can be avoided.

## 1 Introduction

Metamizole is a widely used analgesic agent in many regions around the world, although it has been banned from the market in a few of countries due to a high risk for agranulocytosis [1]. In addition to this rare but life‐threatening adverse event, drug‐induced liver injury (DILI) caused by metamizole has recently gained increasing attention [2, 3, 4, 5, 6, 7, 8]. After sporadic reporting on metamizole causing liver injury, two larger case series were published in Germany reporting on a total of 55 cases with metamizole‐induced liver injury [7, 8]. These publications have raised awareness among clinicians and led to an official Healthcare Professional Communication Letter by the German Federal Institute for Drugs and Medical Devices warning of DILI caused by metamizole. However, despite the increasing knowledge on metamizole DILI, causality assessment can be a challenge. Since metamizole is usually taken together with other medications in patients with pain, acute infections, or undergoing medical procedures, identification of the causative agent in the case of this simultaneous multiple drug intake can pose difficulties. For a variety of medications, typical signature patterns have been proposed that can help to identify the causative medication in the case of polypharmacy and therefore aid to avoid discontinuation of innocent bystander agents [9, 10, 11]. We therefore aimed to further evaluate metamizole DILI cases with the goal of identify characteristic patterns that can help differential diagnosis and causality assessment in the future. In addition, we investigated the predictive value of baseline parameters as well as the time‐dependent dynamic changes of liver parameters in metamizole DILI cases with the aim of rapidly identifying patients at high risk.

## 2 Methods

Patients with acute liver injury (ALI) and suspicion of DILI, who were referred to the LMU Klinikum Munich, Germany, were enrolled in our single‐centre prospective study on patients with acute liver injury and suspected drug‐related causes, which has been described in more detail elsewhere [8]. The data from patients that were enrolled between July 2012 and December 2023 and declared metamizole intake were analysed retrospectively. The procedures were in accordance with the Helsinki Declaration of 1975, as revised in 2013, and the study protocol was approved by the local ethics committee (Project number 55‐13). Written informed consent was obtained from all subjects. ALI was defined according to international consensus criteria [12]: (a) Alanine aminotransferase (ALT) ≥ 5 × upper limit of normal (ULN), (b) alkaline phosphatase (ALP) ≥ 2 × ULN or (c) ALT ≥ 3 × ULN and total bilirubin (TBIL) ≥ 2 × ULN. The type of liver injury was classified using the *R*‐ratio values (ALT/ULN)/(ALP/ULN), with *R* ≥ 5 defining a hepatocellular, *R* ≤ 2 a cholestatic, and 2 < *R* < 5 a mixed‐type injury [12]. Severity of liver injury was graded according to the DILI severity index proposed by the international consensus criteria [12]. According to these guidelines, mild DILI (severity category 1) is defined as elevation of ALT and/or ALP without elevation of TBIL; moderate DILI (severity category 2) as elevation of ALT and/or ALP with TBIL elevation of ≥ 2 × ULN; severe DILI (severity category 3) as additional coagulopathy, ascites, and/or encephalopathy or other organ failure associated with DILI; and fatal DILI (severity category 4) as death or transplantation due to DILI [12]. Hy's law was defined as TBIL > 2 × ULN and ALT > 3 × ULN at the time of onset [13], while jaundice was defined as peak TBIL > 2 mg/dL and coagulopathy as international normalised ratio (INR) ≥ 1.5 at any stage of liver injury. A fatal adverse outcome was defined as death or liver transplantation (LT). Acute liver failure (ALF) was defined according to international criteria: (1) severe ALI characterised by an at least twofold elevation of transaminases, (2) the absence of pre‐existing liver disease, i.e., a disease duration of a maximum of 28 weeks, (3) coagulopathy with an INR ≥ 1.5 in the absence of oral anticoagulants, and (4) hepatic encephalopathy [14].

A thorough hepatological work‐up was performed, including virology testing, liver ultrasound, magnetic resonance imaging (MRI), or computed tomography scan, as well as testing for autoantibodies and metabolic and hereditary liver diseases. The diagnosis of DILI and causality assessment of the causative agent was based on clinical, laboratory, and histopathological findings; the exclusion of alternative causes for liver injury; the Roussel Uclaf Causality Assessment Method (RUCAM) score [15], assessment by the supervising physician; and upon long‐term follow‐up. Forty‐nine of the 80 cases in total were additionally evaluated in a structured expert opinion causality case assessment process. This process included a detailed case presentation and evaluation by at least three independent reviewers with high expertise in the DILI field [16]. The calculation of the RUCAM was performed for each medication with a potential for an association with DILI, meaning that an individual RUCAM score was given for each agent implicated. The total RUCAM score is the sum of points given in seven categories, which are comprised of (1) time to onset, (2) course of liver disease, (3) risk factors, (4) potential DILI by concomitant drugs, (5) exclusion of non‐drug causes of liver injury, (6) previous information on the hepatotoxicity of the drug, and (7) response to rechallenge [17, 18]. The individual points per category range from −3 to +3, and the total achievable score for each adjudicated medication and case ranges from −9 to +14. Likelihood of DILI is rated by the total score as follows: ≤ 0 indicates that the drug is “excluded” as a cause, while 1–2 means that the drug is an “unlikely,” 3–5 a “possible,” 6–8 a “probable,” and > 8 a “highly probable” cause of DILI [17, 18].

Autoimmune hepatitis (AIH) was diagnosed based on laboratory findings, histopathological features, the revised and simplified AIH scores [19, 20] as well as on response to corticosteroids and evaluation during long‐term follow‐up. Biliary obstruction was diagnosed with ultrasound or MRI. Whenever a liver biopsy was performed, histopathological reports were extracted from the patients' medical records in a standardised manner according to a pre‐defined catalogue. Remission was defined as resolution of liver test abnormalities, while chronicity was defined as an elevation of ALT, AST and/or TBIL at least 6 months after liver injury onset.

Data are presented as median and range for continuous and as numbers and percentages for categorical variables. Categorical variables were compared using the Chi‐square test, while non‐parametric tests were applied for continuous variables. If continuous variables were compared between more than two groups, the Kruskal–Wallis test was used, while the Mann–Whitney *U* test was applied for the comparison of variables between two groups only. In case the Kruskal–Wallis test showed a significant difference between the metamizole DILI and two or more control groups, the differences to each comparator group alone were then analysed by the Mann–Whitney *U* test in a second step. *p* ≤ 0.05 was considered statistically significant. For the evaluation of the influence of baseline parameters on outcome, univariate and multivariate logistic regression, the latter with a stepwise backward elimination, were performed. Only variables with *p*‐values < 0.1 in the univariate analysis were included in the multivariate analysis. Significant correlation (*r* ≥ 0.8) between variables was excluded. A receiver‐operating characteristics (ROC) curve was utilised to identify the concordance statistic (*c*‐statistic) of baseline values associated with the diagnosis of the characteristic metamizole DILI pattern and with a fatal adverse outcome in metamizole DILI. Statistical analyses were performed using SPSS (IBM, Armonk, New York, USA, version 29).

## 3 Results

### 3.1 Prevalence of Metamizole DILI

Between 2012 and 2023, 511 patients were included in our prospective study on ALI with a potential drug‐related cause. After thorough work‐up, 324 patients were diagnosed with DILI. Metamizole was associated with liver injury in 61 of those 324 DILI cases, translating into a prevalence of 18.8%. In addition, we identified nine patients with DILI due to an alternative drug despite concomitant metamizole treatment and 10 patients who were initially suspected to have suffered from DILI due to metamizole alone or due to metamizole and concomitant drugs but who were later diagnosed with AIH.

### 3.2 Clinical Characteristics

The clinical characteristics of patients with metamizole DILI in comparison to control cases (comprised of non‐metamizole DILI [*n* = 9] and AIH [*n* = 10] patients with concomitant metamizole intake) can be reviewed in Table 1. There were no significant differences regarding age, body mass index (BMI), or sex between the three groups. The majority of metamizole DILI patients were female (68.9%) and middle‐aged (median: 44 years). The median daily dosage of metamizole was significantly lower in the metamizole DILI group when compared to the non‐metamizole DILI group (1000 mg vs. 2000 mg, *p* = 0.011) but comparable to the AIH patients (both 1000 mg, non‐significant [ns]; Table 1). The latency from the start of metamizole intake until the onset of liver injury was comparable between all three groups (41 days [range: 2–674 days] in patients with metamizole DILI vs. 38 days [1–515] and 52 days [3–113] in the control groups, respectively; *p* = 0.827). Thirty‐eight patients with metamizole DILI (62.2%) had discontinued metamizole before the appearance of liver injury, while this was the case in six non‐metamizole DILI (66.6%) and five AIH cases (55.5%). Latency from the end of drug intake until DILI recognition was comparable in metamizole DILI and the control groups as well (Table 1). In the metamizole DILI group, the median RUCAM for metamizole was 6 (range 2–11), and 98.4% had a RUCAM of 3 or higher, indicating that metamizole DILI was at least a possible cause for liver injury.

**TABLE 1:** Clinical characteristics in patients with metamizole DILI and controls.

	Metamizole DILI (*n* = 61)	Non‐metamizole DILI (*n* = 9)	AIH (*n* = 10)	*p*
Clinical characteristics				
Age (years)	44 (19–84)	36 (22–75)	54 (33–73)	0.198
Body mass index (kg/m^2^)	24.1 (18.1–37.9)	22.2 (16.8–37.7)	31.2 (20.5–58.8)	0.062
Female	42 (68.9%)	5 (55.6%)	7 (70.0%)	0.717
Average daily dosage metamizole (mg)	1000 (71–4000)	2000 (1500–4000)	1000 (1000–2250)	0.039* (0.011*, 0.068)
Latency from start of drug intake until onset of DILI (days)	41 (2–674)	38 (1–515)	52 (3–113)	0.827
Latency from last day of intake until onset of DILI (days)^a^	13 (1–106)	25 (1–96)	15 (8–41)	0.572
RUCAM metamizole^b^	6 (2–11)	4 (2–6)	6 (0–9)	< 0.001* (< 0.001*, 0.218)
RUCAM metamizole ≥ 3	60 (98.4%)	8 (88.9%)	9 (90.0%)	0.203
Usage of co‐medications in the context of DILI				
No concomitant medication	2 (3.3%)	0 (0.0%)	0 (0.0%)	0.081
Use of co‐medication but without compatible time to onset	18 (29.5%)	0 (0.0%)	0 (0.0%)	
Use of co‐medication with compatible time to onset	41 (67.2%)	9 (100.0%)	10 (100.0%)	
Latency concomitant medication	30 (2–279)	26 (6–76)	33 (14–109)	0.420
RUCAM of the concomitant medication^b^	5 (2–8)	6 (4–9)	5 (0–7)	0.268
AST at onset (×ULN)	26.9 (1.1–115.3)	4.4 (1.4–44.3)	17.4 (5.5–117.5)	0.032* (0.009*, 0.523)
ALT at onset (×ULN)	36.5 (3.8–138.5)	6.0 (0.8–88.3)	26.3 (9.9–82.3)	0.019* (0.007*, 0.259)
ALP at onset (×ULN)	1.6 (0.6–13.7)	2.1 (0.5–4.9)	1.8 (0.7–5.2)	0.990
TBIL at onset (×ULN)	8.2 (0.2–24.3)	0.9 (0.2–5.8)	7.4 (0.7–22.5)	0.009* (0.003*, 0.789)
INR at onset	1.4 (0.8–6.1)	1.1 (0.9–1.4)	1.4 (0.9–2.0)	0.052
MELD at onset	19 (6–35)	8 (6–14)	21 (6–26)	0.016* (0.005*, 0.808)
*R*‐ratio	20.5 (0.4–83.1)	5.1 (0.2–136.3)	10.7 (2.7–80.9)	0.013* (0.007*, 0.126)
Pattern of liver injury based on *R*‐ratio^c^				
Hepatocellular	51 (83.6%)	5 (55.6%)	9 (90.0%)	0.037*
Mixed	7 (11.5%)	1 (11.1%)	1 (10.0%)	
Cholestatic	3 (4.9%)	3 (33.3%)	0 (0.0%)	
ANA positivity	44 (72.1%)	5 (55.6%)	7 (70.0%)	0.834
ANA titre	1:100 (0–1:25 600)	1:100 (1:100–1:1600)	1:200 (1:100–1:3200)	0.415
AMA positivity	15 (25.0%)	0 (0.0%)	2 (20.0%)	0.404
IgG (g/dL)	12.9 (5.3–24.7)	9.7 (4.8–16.5)	16.6 (10.4–28.0)	0.019* (0.219, 0.017*)
IgG>ULN	15 (24.6%)	1 (11.1%)	6 (60.0%)	0.066
Recurrence of liver injury upon rechallenge	9 (14.8%)	0 (0.0%)	0 (0.0%)	
With metamizole alone	5 (8.2%)	0 (0.0%)	0 (0.0%)	0.532
With metamizole and concomitant medication	4 (6.6%)	0 (0.0%)	0 (0.0%)	
Severity and outcome				
DILI severity index^d^	3 (1–4)	1 (1–3)	3 (1–4)	0.003* (< 0.001*, 0.500)
Hy's law positive	43 (70.5%)	3 (33.3%)	7 (70.0%)	0.086
Jaundice at peak TBIL levels	53 (86.9%)	4 (44.4%)	8 (80.0%)	0.010*
Coagulopathy	32 (52.5%)	1 (11.1%)	5 (50.0%)	0.067
Acute liver failure^e^	16 (26.2%)	0 (0.0%)	3 (30.0%)	0.199
Corticosteroid treatment	23 (37.7%)	2 (22.2%)	9 (90.0%)	0.003*
Remission	47 (77.0%)	6 (66.7%)	7 (70.0%)	0.525
Time to remission (weeks)	10 (3–168)	3 (2–45)	20 (14–23)	0.012* (0.125, 0.021)
Follow‐up (weeks)	29 (1–470)	14 (5–204)	69 (6–277)	0.090

*Note:* Categorical variables are presented as numbers and percentages (*n* (%)). Continuous variables are presented as median (range). Metamizole DILI cases were identified based on clinical, laboratory, and histopathological findings, the exclusion of alternative diagnoses, assessment by the physician in charge, and upon long‐term follow‐up. In addition, the Roussel Uclaf Causality Assessment Method (RUCAM) [17, 18] was applied, and a structured expert opinion causality case assessment process was performed [16]. The Kruskal–Wallis test was applied for the assessment of significance of continuous variables. If a statistical significance was observed between the metamizole DILI and both comparator groups, the individual *p* values comparing metamizole DILI versus non‐metamizole DILI and AIH controls, respectively, were determined by the Mann–Whitney *U* test and are stated in brackets.

Abbreviations: AIH, autoimmune hepatitis; ALP, alkaline phosphatase; ALT, alanine aminotransferase; AMA, anti‐mitochondrial antibodies; ANA, antinuclear antibodies; ASMA, anti‐smooth muscle antibodies; AST, aspartate aminotransferase; DILI, drug‐induced liver injury; IgG, immunoglobulin G; INR, international normalised ratio; MELD, model for end‐stage liver disease; RUCAM, Roussel Uclaf Causality Assessment Method; TBIL, total bilirubin; ULN, upper limit of normal.

^a^ Metamizole was discontinued before the onset of liver injury in 38 metamizole DILI, 6 non/metamizole DILI, and 5 AIH cases.

^b^ The interpretation of the final RUCAM score is: ≤ 0: excluded; 1–2: unlikely; 3–5: possible; 6–8: probable; and > 8: highly probable [17, 18].

^c^ The *R*‐ratio is defined as (ALT/ULN)/(ALP/ULN), with *R* ≥ 5 defining a hepatocellular, *R* ≤ 2 a cholestatic and 2 < *R* < 5 a mixed type of injury.

^d^ According to international consensus guidelines [12].

^e^ Acute liver failure was defined as a severe acute liver injury with a maximum duration of 28 weeks as well as the presence of coagulopathy and encephalopathy.

* Statistical significance (*p* ≤ 0.05).

In the metamizole DILI cohort, only two patients did not have any concomitant medications, while most patients (67.2%) had been taking other remedies at the same time as metamizole. In addition, 29.5% were under treatment with co‐medications; however, with no compatible time to onset. Most patients concomitantly used non‐steroidal anti‐inflammatory drugs (NSAID, *n* = 27, 44.3%) or antimicrobial drugs (*n* = 17, 27.9%, data not shown). Concomitant medication with compatible timing to onset was used by every patient in the control groups, and the RUCAM scores for the concomitant medications were comparable between all three groups (Table 1).

Interestingly, significant differences regarding some baseline characteristics were mostly observed between metamizole DILI and non‐metamizole DILI patients, while the clinical features were widely comparable between metamizole DILI and AIH patients. As such, the median RUCAM for metamizole was significantly higher in metamizole DILI than in non‐metamizole DILI cases (6 [range: 2–11] vs. 4 [range: 2–6], *p* = 0.009), but similar in the AIH patients (6 [range: 2–11] vs. 6 [range: 0–9], *p* = 0.218; Table 1). Moreover, patients with metamizole DILI showed higher elevation of ALT and aspartate aminotransferase (AST) when compared to non‐metamizole DILI patients (ALT: 36.5 × ULN vs. 6.0 × ULN, *p* = 0.007; AST: 26.9 × ULN vs. 4.4 × ULN, *p* = 0.009), while ALT and AST levels were comparable to those observed in AIH patients (ALT: 36.5 × ULN vs. 26.3 × ULN, *p* = 0.259; AST: 26.9 × ULN vs. 17.4 × ULN, *p* = 0.523; Table 1). TBIL was also significantly higher in metamizole DILI patients when compared to the non‐metamizole DILI controls (8.2 × ULN vs. 0.9 × ULN, *p* = 0.003), yet a relevant increase of ALP was neither observed in metamizole DILI nor in control cases (Table 1). Most patients with metamizole DILI and AIH presented with a hepatocellular type of injury (83.6% and 90.0%), while only 55.6% of the non‐metamizole‐DILI cases did (*p* = 0.037). Accordingly, the *R*‐ratio at onset was significantly higher in patients with metamizole DILI when compared to non‐metamizole DILI controls (20.5 vs. 5.1, *p* = 0.007). Immunoglobulin G (IgG) levels were significantly higher in AIH patients when compared to metamizole DILI and non‐metamizole DILI cases (16.6 g/dL vs. 12.9 g/dL and 9.7 g/dL, respectively, *p* = 0.017 and *p* = 0.009); however all three groups showed comparable rates of antinuclear antibodies (ANA) positivity (72.1% vs. 55.6% vs. 70.0%, *p* = 0.834; Table 1).

The DILI severity index, as well as the proportions of patients with Hy's law positivity, coagulopathy, and ALF, were similar in metamizole DILI and AIH patients but higher than in the non‐metamizole DILI group (Table 1). Significantly more patients with metamizole DILI developed jaundice throughout the liver injury episode when compared to the non‐metamizole DILI controls (86.9% vs. 44.4%; Table 1). Despite the higher severity in metamizole DILI patients, comparable remission rates were observed in all three groups (77.0% vs. 66.7% and 70.0%, *p* = 0.525). Time to remission, however, was significantly shorter in metamizole DILI patients than in AIH patients (10 vs. 20 weeks, *p* = 0.021), while there was a tendency towards a longer time to remission when compared to non‐metamizole DILI cases (10 vs. 3 weeks), although this difference was not statistically significant (*p* = 0.125, Table 1).

Throughout the whole cohort of metamizole DILI cases, 18 of the 61 cases scored a RUCAM below 6 (29.5%), meaning that metamizole was an “unlikely” or “possible” cause of liver injury, while 43 patients (70.5%) scored 6 or higher, indicating metamizole was a “probable” or “highly probable” cause of DILI. However, apart from a higher INR at onset and peak levels in patients with a RUCAM of 3–5, no differences were observed between metamizole DILI patients with RUCAM < 6 or ≥ 6 regarding liver parameters, neither at baseline nor at peak levels, nor regarding the evolution of liver parameters over time (Table S1).

### 3.3 Recurrence of Liver Injury Upon Rechallenge—A Common Phenomenon in Metamizole DILI Patients

Nine patients from the metamizole DILI group (14.8%) presented with another episode of DILI after re‐challenge with metamizole alone (*n* = 5, 8.2%) or metamizole and concomitant medication (*n* = 4, 6.6%; Table 1). One patient, for instance, presented at our centre with abdominal pain for 7 days and jaundice for 5 days. Laboratory sampling at presentation showed marked elevation of ALT (34.4 × ULN), AST (41.2 × ULN) and TBIL (9.1 × ULN) as well as coagulopathy (INR 1.9). The patient reported the intake of ibuprofen and paracetamol, which she had started 23 days and stopped 7 days before presentation, as well as metamizole and amoxicillin/clavulanic acid, which she had started 23 days and stopped 17 days before presentation. The hepatological work‐up excluded viral hepatitis, biliary obstruction, and metabolic as well as hereditary liver diseases. When the medical history was re‐evaluated closely, it became apparent that the patient had suffered from ALF 14 years earlier. Initial liver function tests back then had been similar, with highly elevated ALT, AST, and TBIL (40.9 × ULN, 33.7 × ULN and 16.1 × ULN) as well as coagulopathy (INR = 3.5). Previously, alternative diagnoses had been excluded as well. In addition, liver biopsy had shown subacute liver dystrophy with mixed cell type inflammation and ductular proliferation but no fibrosis nor elevation of iron or copper and was therefore rated as most likely DILI. During that episode, liver injury was associated with cyproterone acetate; however, at thorough re‐interrogation, the patient reported intake of metamizole in the weeks before that episode as well. In addition, she had used cyproterone acetate after this DILI episode again without recurrence of liver injury, the negative rechallenge therefore excludes causality with this agent. Rapidly after both liver injury incidents, liver function tests completely normalised. However, the patient once again presented with ALI 22 months after the second episode. Due to suffering from a traumatic brain injury following a bike accident, she had received amoxicillin/clavulanic acid, ibuprofen, and once again metamizole. Four weeks later, a laboratory control showed a marked elevation of ALT and AST (62.9 × ULN and 57.1 × ULN) as well as increasing levels of TBIL (1.9 × ULN) and INR (1.5). Amoxicillin/clavulanic acid and metamizole had been started 29 days and stopped 22 days before that presentation; ibuprofen had been started 29 days before and had been taken intermittently as needed until the day of presentation. Liver biopsy was re‐performed, showing eosinophilic hepatitis with signs of interface hepatitis as well as single‐cell necrosis, and was rated as most likely being associated with DILI. After onset of this last episode, AST, and ALT rapidly decreased, while TBIL peaked 12 days after presentation. Fifty‐eight days later, liver parameters had completely normalised and did not show another increase in the long‐term follow‐up. The patient reported that she intermittently was taking ibuprofen again after the third episode; metamizole and amoxicillin/clavulanic acid were never used again. Due to the spontaneous and rapid normalisation and the lack of signs for chronic liver disease in the repeatedly performed liver biopsy, AIH could be excluded in this case, leaving DILI as the only possible diagnosis. The only drug that was taken at all three time points was metamizole, strongly suggesting the causal relationship in this case.

### 3.4 The Characteristic Metamizole DILI Signature

**FIGURE 1:**
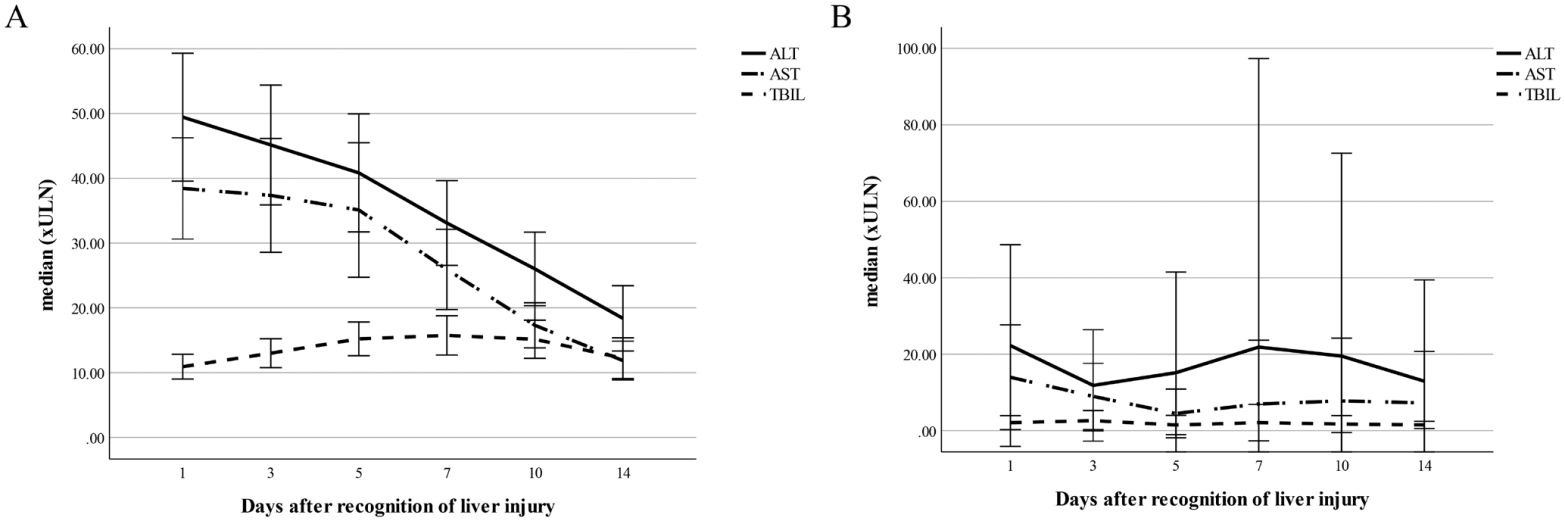
Evolution of liver parameters in patients with the characteristic metamizole DILI signature pattern versus non‐metamizole DILI. The graph demonstrates the mean values (with the 95% CI) for ALT, AST and TBIL of the patients with the characteristic metamizole DIIL signature pattern (*n* = 43; A) versus the patients with non‐metamizole DILI (*n* = 9; B). ALT, alanine aminotransferase; AST, aspartate aminotransferase; CI, confidence interval; DILI, drug‐induced liver injury; TBIL, total bilirubin.

A characteristic clinical pattern was noticed in a large proportion of patients with metamizole DILI, which was also apparent in the case presented above: High elevation of AST and ALT at the time of DILI recognition with a rapid decline shortly after onset and a relatively high initial TBIL value, which however, continues to increase after DILI recognition (Figure 1A). As demonstrated in Figure 1B, non‐metamizole DILI cases rather show a secondary increase of ALT and AST and no secondary TBIL peak. Forty‐three of the 61 metamizole DILI patients (70.5%) fit this clinical picture. When the patients with the characteristic metamizole DILI signature pattern were compared to a control group comprised of metamizole DILI cases without this clinical picture (*n* = 18), patients with non‐metamizole DILI despite metamizole intake (*n* = 9) and AIH patients with prior metamizole intake (*n* = 10), significantly higher AST, ALT, TBIL, and INR levels, as well as MELD scores were seen at onset and peak values in the patients with the characteristic metamizole DILI pattern (Table 2).

**TABLE 2:** Comparison of laboratory evolution between patients with the characteristic metamizole DILI signature pattern and control subgroups.

	Metamizole DILI signature pattern (*n* = 43)	Other metamizole DILI (*n* = 18)	Non‐metamizole DILI (*n* = 9)	AIH (*n* = 10)	*p*
At the time of DILI recognition					
AST (×ULN)	36.9 (2.5 to 115.3)	10.9 (1.1 to 32.3)	4.4 (1.3 to 44.3)	17.4 (5.5 to 117.5)	< 0.001* (< 0.001*, < 0.001*, 0.124)
ALT (×ULN)	43.6 (3.9 to 138.5)	19.1 (3.8 to 62.0)	6.0 (0.8 to 88.3)	26.3 (9.9 to 82.3)	< 0.001* (0.001*, < 0.001*, 0.064)
ALP (×ULN)	1.8 (0.9 to 13.7)	1.6 (0.6 to 5.4)	2.1 (0.5 to 4.9)	1.8 (0.7 to 5.2)	0.455
TBIL (×ULN)	9.8 (0.2 to 24.3)	1.3 (0.4 to 13.5)	0.9 (0.2 to 5.8)	7.4 (0.7 to 22.5)	< 0.001* (< 0.001*, < 0.001*, 0.407)
INR	1.5 (0.8 to 6.1)	1.1 (0.9 to 1.7)	1.1 (0.9 to 1.4)	1.4 (0.9 to 2.0)	< 0.001* (< 0.001*, 0.001*, 0.236)
MELD	21 (6 to 35)	7 (6 to 21)	8 (6 to 14)	21 (6 to 26)	< 0.001* (< 0.001*, < 0.001*, 0.435)
*R*‐ratio^a^	24.3 (0.4 to 83.1)	16.6 (2.2 to 38.7)	5.1 (0.2 to 136.3)	10.7 (2.7 to 80.9)	0.010* (0.106, 0.003*, 0.062)
Peak values					
AST (×ULN)	44.1 (4.0 to 115.3)	10.9 (1.7 to 69.4)	7.2 (1.4 to 44.3)	20.6 (5.5 to 117.5)	< 0.001* (< 0.001*, < 0.001*, 0.124)
ALT (×ULN)	48.9 (6.3 to 145.5)	21.2 (5.9 to 105.9)	8.2 (1.0 to 88.3)	26.3 (11.0 to 82.3)	< 0.001* (< 0.001*, < 0.001*, 0.062)
ALP (×ULN)	2.2 (1.0 to 15.4)	1.7 (1.0 to 5.4)	2.5 (0.5 to 6.9)	2.8 (1.2 to 6.9)	0.168
TBIL (×ULN)	20.2 (2.8 to 34.5)	2.1 (0.5 to 15.5)	1.2 (0.2 to 6.0)	17.8 (1.3 to 25.1)	< 0.001* (< 0.001*, < 0.001*, 0.166)
INR	1.7 (0.9 to 8.0)	1.1 (0.9 to 1.7)	1.0 (0.9 to 1.5)	1.7 (0.9 to 3.9)	< 0.001* (< 0.001*, < 0.001*, 0.406)
MELD	22 (13 to 40)	10 (6 to 21)	9 (6 to 16)	23 (6 to 38)	< 0.001* (< 0.001*, < 0.001*, 0.610)
*R*‐ratio^a^	29.6 (0.4 to 91.5)	17.6 (2.8 to 116.1)^b^	12.5 (0.1 to 136.3)	14.1 (2.7 to 80.9)	0.025* (0.038*, 0.043*, 0.033*)
Time‐dependent dynamic changes of liver parameters^c^					
ΔAST day 1–3	−2.7 (−36.3 to 13.8)	−4.9 (−19.9 to 4.2)	1.1 (−19.4 to 5.6)	−1.2 (−51.3 to 1.9)	0.895
ΔAST day 1–7	−17.0 (−76.4 to 25.7)	−3.0 (−22.8 to 20.4)	−2.3 (−20.1 to 2.4)	−3.1 (−89.7 to 20.5)	0.311
ΔALT day 1–3	−6.9 (−59 to 15.5)	−5.3 (−13.2 to 8.3)	0.9 (−12.8 to 2.0)	−5.2 (−25.1 to 0.0)	0.451
ΔALT day 1–7	−10.4 (−79.2 to 23.1)	−2.6 (−131 to 8.3)	−3.4 (−31.5 to 4.3)	−10.6 (−53.6 to 10.6)	0.460
ΔTBIL day 1–3	1.8 (−5.2 to 12.4)	0.0 (−1.1 to 1.1)	0.0 (−0.4 to 0.9)	−0.7 (−6.5 to 2.0)	< 0.001* (0.003*, 0.011*, 0.002*)
ΔTBIL day 1–7	4.5 (−8.0 to 17.3)	−0.3 (−3.4 to 2.8)	−0.1 (−1.4 to −0.1)	0.7 (−4.8 to 14.3)	0.002* (0.002*, 0.022*, 0.111)

*Note:* Variables are presented as median (range). The Kruskal–Wallis test was applied for the assessment of the significance of continuous variables. If a statistical significance was observed between the metamizole DILI signature pattern and all three comparator groups, the individual *p*‐values comparing the metamizole DILI signature pattern groups versus other metamizole DILI, non‐metamizole DILI, and AIH controls, respectively, were determined by the Mann–Whitney *U* test and are stated in brackets accordingly.

Abbreviations: AIH, autoimmune hepatitis; ALP, alkaline phosphatase; ALT, alanine aminotransferase; AST, aspartate aminotransferase; DILI, drug‐induced liver injury; INR, international normalised ratio; MELD, model for end‐stage liver disease; TBIL, total bilirubin; ULN, upper limit of normal.

^a^ The *R*‐ratio is defined as (ALT/ULN)/(ALP/ULN), with *R* ≥ 5 defining a hepatocellular, *R* ≤ 2 a cholestatic, and 2 < *R* < 5 a mixed types of injury.

^b^ The peak *R*‐ratio was calculated on the date of peak ALT for hepatocellular and mixed types of injury and on the date of peak ALP for cholestatic types of injury.

^c^ Under this section, the time‐based changes of the respective liver parameters from day 1 to day 3 or day 7 expressed as fold ULN are shown.

* Statistical significance (*p* ≤ 0.05).

The differences regarding liver parameter elevation were most pronounced between patients with the characteristic metamizole DILI pattern when compared to other metamizole DILI or non‐metamizole DILI cases, while baseline and peak liver parameters were not significantly different when compared to AIH patients. One of the only two features that was significantly higher in patients with the characteristic metamizole DILI pattern when compared to each control group alone, including AIH patients, was the *R*‐ratio at peak ALT or ALP levels (29.6 vs. 17.6 vs. 12.5 and 14.1 in patients with the characteristic metamizole DILI pattern, other metamizole DILI, non‐metamizole DILI, and AIH cases, respectively, *p* = 0.025; Table 2). In addition, the time‐based change of TBIL from day 1 to day 3 expressed as fold ULN (ΔTBIL3) was the only other factor with a significant difference when compared to each control group alone (1.8 × ULN vs. 0.0 × ULN, 0.0 × ULN, and −0.7 × ULN, *p* < 0.001; Table 2). Even when all metamizole cases were compared to controls, ΔTBIL3 remained statistically different (+1.1 × ULN vs. −0.1; *p* = 0.022, data not shown).

**FIGURE 2:**
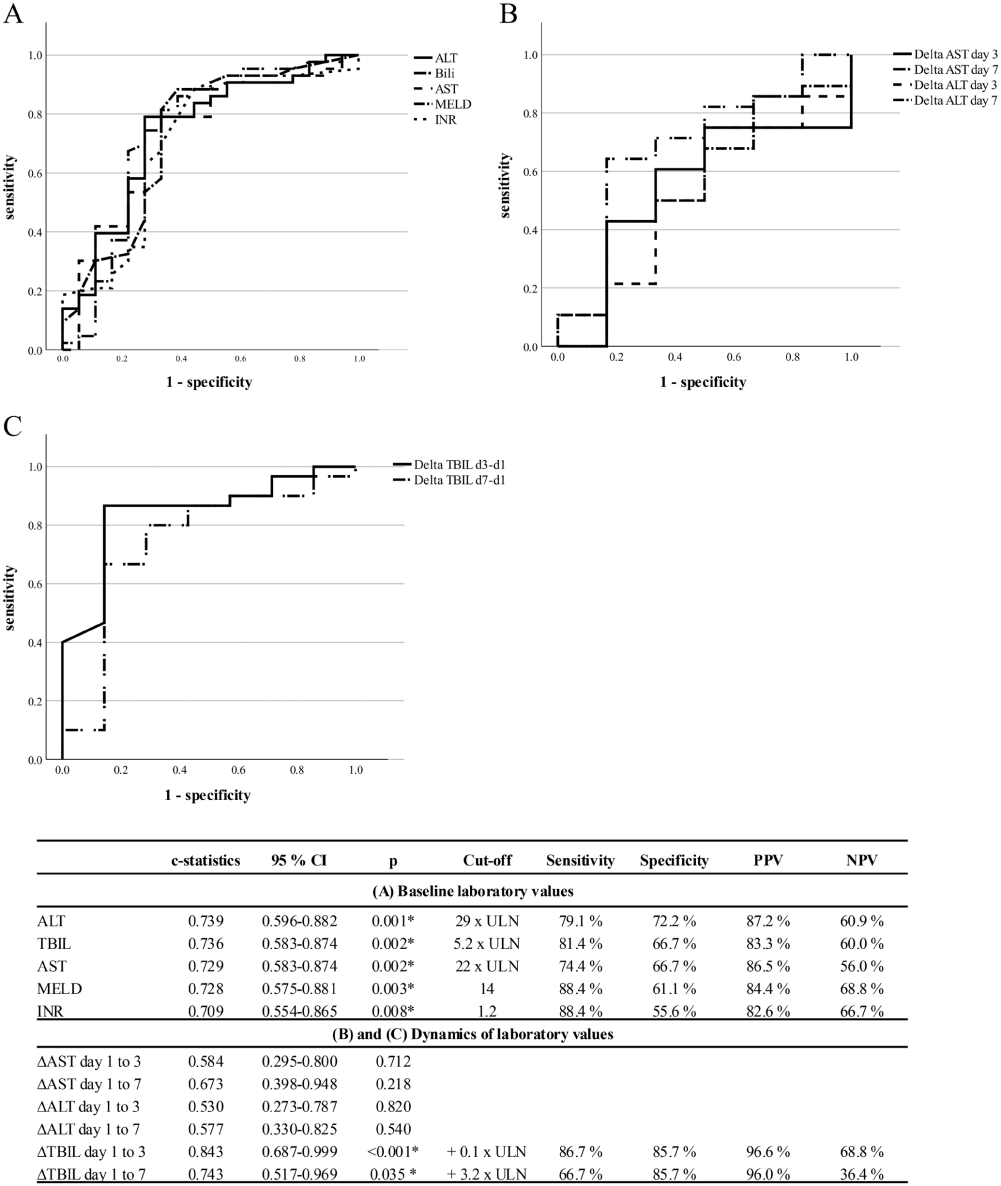
ROC analysis for laboratory parameters at the time of clinical presentation and time‐dependent dynamic changes of liver parameters with regards diagnosis metamizole DILI with the characteristic signature pattern. Shown are the *c*‐statistics, sensitivity and specificity of (A) baseline parameters and scores at the time of clinical presentation, (B) the time‐dependent dynamic changes of AST, ALT and (C) the time‐dependent dynamic changes of TBIL regarding the diagnosis of metamizole DILI with the characteristic metamizole DILI signature pattern versus controls (as defined by non‐metamizole DILI and AIH). The cut‐offs were determined by ROC curve analysis and Youden's index. ALT, alanine aminotransferase; AST, aspartate aminotransferase; CI, confidence interval; *c*‐statistics, concordance statistic; DILI, drug‐induced liver injury; INR, international normalised ratio; MELD, model for end‐stage liver disease; NPV, negative predictive value; PPV, positive predictive value; ROC, receiver of the operator characteristic; TBIL, total bilirubin; ULN, upper limit of normal. * statistical significance (*p* < = 0.05).

Figure 2 demonstrates the ROC curve and *c*‐statistics for baseline TBIL, MELD, ALT, AST, and INR regarding the diagnosis of metamizole DILI with the signature pattern in comparison to non‐metamizole DILI and AIH cases despite metamizole intake: The highest *c*‐statistics were observed for ALT and TBIL at onset (0.74, respectively). For both baseline parameters, high positive predictive values (PPV) were observed (ALT: 87.2% at a cut‐off of 29 × ULN; TBIL: 83.3% at a cut‐off of 5.2 × ULN), while the highest sensitivity was observed for INR at a cut‐off of 1.2 (88.4%; Figure 2). The time‐dependent dynamic changes of liver parameters, in particular ΔTBIL3, showed an even better discriminatory power with a *c*‐statistic of 0.84. At a cut‐off of +0.1 × ULN above baseline 3 days after DILI recognition, metamizole DILI could be distinguished from controls with a remarkable PPV of 96.6% as well as high sensitivity and specificity (86.7% and 85.7%, respectively; Figure 2).

Regarding the clinical characteristics at baseline, there was no feature that showed a significant difference between patients with the metamizole DILI signature pattern and each control group alone (Table 3). The average daily dosage of metamizole, however, was higher in the non‐metamizole DILI cases, while the RUCAM was lower in this group when compared to the patients with the characteristic metamizole DILI injury pattern (Table 3). When the clinical characteristics were compared between patients with the characteristic metamizole DILI signature pattern and other metamizole DILI cases, the only parameter that was significantly different was IgG (13.7 g/dL vs. 10.8 g/dL, *p* = 0.047; Table 3).

**TABLE 3:** Comparison of clinical characteristics and outcome in patients with the characteristic metamizole DILI signature pattern and controls.

	Metamizole DILI signature pattern (*n* = 43)	Other metamizole DILI (*n* = 18)	Non‐metamizole DILI (*n* = 9)	AIH (*n* = 10)	*p*
Age (years)	43 (19–79)	50 (20–84)	36 (22–75)	54 (33–73)	0.263
Body mass index (kg/m^2^)	24.7 (18.1–37.9)	23.4 (18.9–30.6)	22.2 (16.8–37.7)	31.2 (20.5–58.8)	0.077
Female	29 (67.4%)	13 (72.2%)	5 (55.6%)	7 (70.0%)	0.850
Average daily dosage metamizole (mg)	1000 (71–4000)	1250 (500–3000)	2000 (1500–4000)	1000 (1000–2250)	0.042* (0.188, 0.007*, 0.483)
Latency from start of drug intake until onset of DILI (days)	48 (2–674)	31 (2–102)	38 (1–515)	52 (3–113)	0.190
Latency from last day of intake until onset of DILI (days)^a^	17 (1–106)	9 (1–48)	25 (1–96)	15 (8–41)	0.685
RUCAM metamizole^b^	6 (2–9)	7 (4–11)	4 (2–6)	6 (0–9)	0.001* (0.379, < 0.001*, 0.322)
RUCAM metamizole ≥ 3	42 (97.7%)	18 (100.0%)	8 (88.9%)	9 (90.0%)	0.337
Latency concomitant medication	31 (4–279)	15 (2–102)	26 (6–76)	33 (14–109)	0.154
RUCAM of the concomitant medication^b^	5 (2–8)	5 (3–7)	6 (4–9)	5 (0–6)	0.452
Pattern of liver injury based on *R* value^c^					
Hepatocellular	37 (86.0%)	14 (77.8%)	5 (55.6%)	9 (90.0%)	0.033*
Mixed	3 (7.0%)	4 (22.2%)	1 (11.1%)	1 (10.0%)	
Cholestatic	3 (7.0%)	0 (0.0%)	3 (33.3%)	0 (0.0%)	
ANA positivity	30 (69.8%)	14 (77.8%)	5 (55.6%)	7 (70.0%)	0.716
ANA titre	1:100 (0–1:25 600)	1:100 (0–1:3200)	1:100 (1:100–1:1600)	1:200 (1:100–1:3200)	0.659
AMA positivity	11 (25.6%)	4 (22.2%)	0 (0.0%)	2 (20.0%)	0.612
IgG (g/dL)	13.7 (6.5–27.6)	10.8 (5.3–24.3)	9.7 (4.8–16.5)	16.6 (10.4–28.0)	0.008* (0.047*, 0.177, 0.061)
IgG>ULN	12 (27.9%)	3 (16.7%)	1 (11.1%)	6 (60.0%)	0.116
Severity and outcome					
DILI severity index^d^	3 (2–4)	2 (1–3)	1 (1–3)	3 (1–4)	< 0.001* (< 0.001*, < 0.001*, 0.108)
Hy's law positive	38 (88.4%)	5 (27.8%)	3 (33.3%)	7 (70.0%)	< 0.001*
Jaundice at peak TBIL levels	43 (100.0%)	10 (55.6%)	4 (44.4%)	8 (80.0%)	< 0.001*
Coagulopathy	31 (72.1%)	1 (5.6%)	1 (11.1%)	5 (50.0 5)	< 0.001*
Acute liver failure^e^	16 (37.2%)	0 (0.0%)	0 (0.0%)	3 (30.0%)	0.005*
Corticosteroid treatment	19 (44.2%)	4 (22.2%)	2 (22.2%)	9 (90.0%)	0.003*
Remission	32 (74.4%)	15 (83.3%)	6 (66.7%)	7 (70.0%)	0.441
Time to remission (weeks)	10 (3–168)	10 (3–20)	3 (2–45)	20 (14–23)	0.025* (0.449, 0.095, 0.044*)

*Note:* Categorical variables are presented as numbers and percentages (*n* (%)). Continuous variables are presented as median (range). The Kruskal–Wallis test was applied for the assessment of the significance of continuous variables. If a statistical significance was observed between the metamizole DILI signature pattern and all three comparator groups, the individual *p*‐values comparing the metamizole DILI signature pattern groups versus other metamizole DILI, non‐metamizole DILI, and AIH controls, respectively, were determined by the Mann–Whitney *U* test and are stated in brackets.

Abbreviations: AIH, autoimmune hepatitis; AMA, anti‐mitochondrial antibodies; ANA, antinuclear antibodies; DILI, drug‐induced liver injury; IgG, immunoglobulin G; INR, international normalised ratio; RUCAM, Roussel Uclaf Causality Assessment Method; TBIL, total bilirubin; ULN, upper limit of normal.

^a^ In case metamizole was discontinued before the onset of liver injury.

^b^ The interpretation of the RUCAM score is: ≤ 0: excluded; 1–2: unlikely; 3–5: possible; 6–8: probable; and > 8: highly probable [17, 18].

^c^ The *R*‐ratio is defined as (ALT/ULN)/(ALP/ULN), with *R* ≥ 5 defining a hepatocellular, *R* ≤ 2 a cholestatic, and 2 < *R* < 5 a mixed type of injury.

^d^ According to international consensus guidelines [12].

^e^ Acute liver failure was defined as a severe acute liver injury with a maximum duration of 28 weeks as well as the presence of coagulopathy and encephalopathy.

* Statistical significance (*p* ≤ 0.05).

Regarding severity and outcome, patients with the characteristic metamizole DILI injury pattern showed a higher DILI severity index than other metamizole and non‐metamizole DILI cases (3 vs. 2 and 1, *p* < 0.001, respectively) but not when compared to AIH patients (Table 3). In addition, patients with the characteristic metamizole DILI signature pattern were more likely to be Hy's law positive upon presentation, develop jaundice or coagulopathy, and to progress to ALF. However, despite higher severity, remission rates did not differ significantly between the four groups (Table 3).

### 3.5 Histological Analysis

**FIGURE 3:**
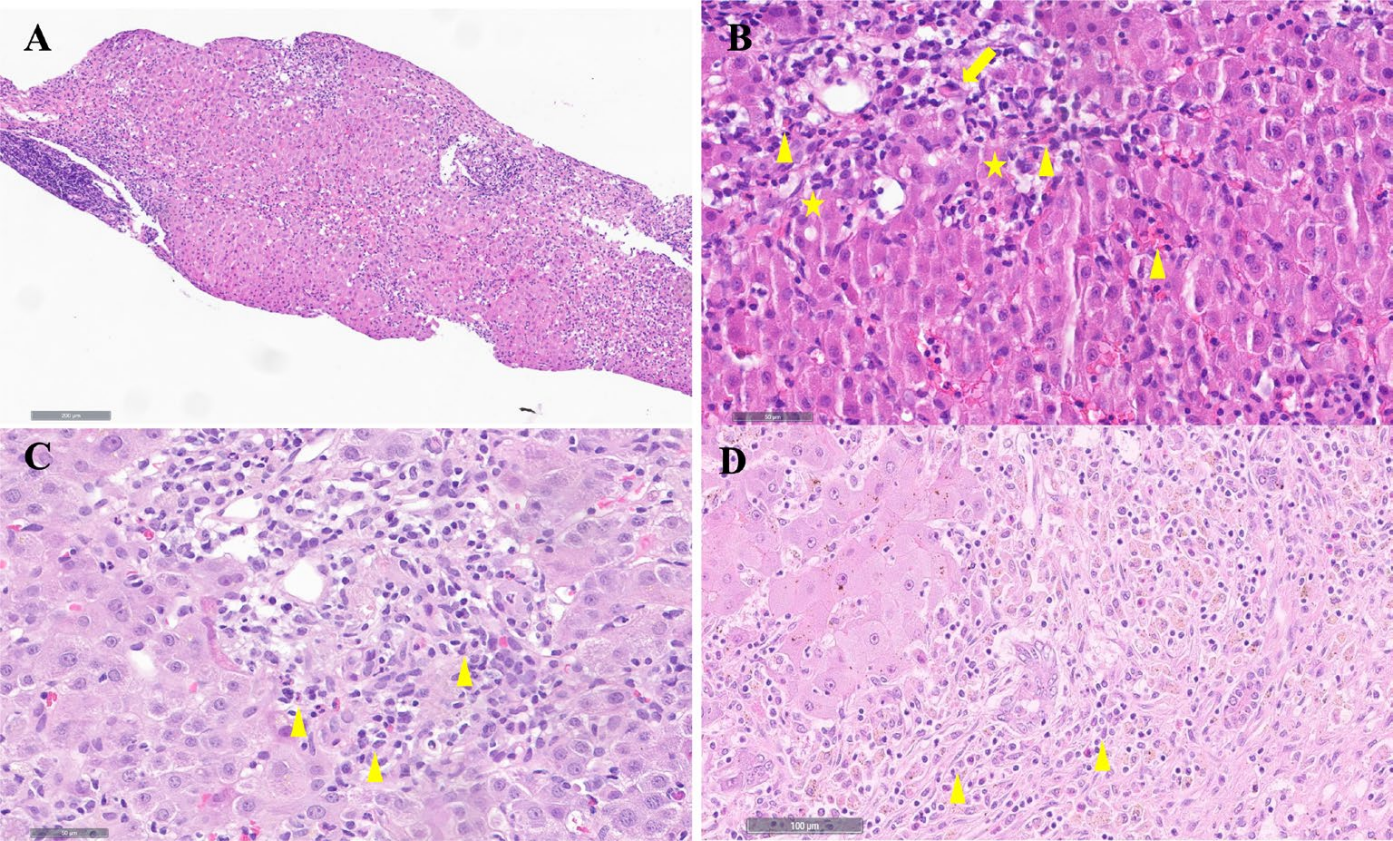
Representative histological images of patients with metamizole DILI (H&E). The slides represent typical findings in metamizole DILI. (A, B) Hepatitis with predominant eosinophilic infiltration (arrowhead) and marked interface hepatitis (asterisk), single cell necrosis (arrow) and mild lipofuscinosis. (C) Eosinophilic hepatitis (arrowhead) with interface hepatitis, single as well as grouped cell necrosis and mild canalicular cholestasis. (D) Subacute confluent liver necrosis with lymphocytic infiltration (arrowhead) in a patient with acute liver failure, the necrosis is mostly localised in zone 1 and 2.

Forty‐five of the 61 patients (73.7%) with metamizole DILI received liver biopsy sampling. All of the patients presented with inflammatory infiltrates, which were predominately of a mixed cell type or comprised of lymphocytes and plasma cells (Table 4). Inflammatory infiltrates were mainly localised in the portal and/or intralobular areas. 44.4% presented with interface hepatitis, while plasma cells were detected in 48.9%. In addition, eosinophilic cells were observed in 68.9%. Cholestasis was seen in 42.2%, which was mainly canalicular and hepatocellular. Necrosis was highly present (84.4%), with 28.2% showing severe confluent necrosis (Table 4). Representative histological slides showing eosinophilic hepatitis, interface hepatitis, and extensive necrosis can be reviewed in Figure 3.

**TABLE 4:** Histopathological characteristics in patients with metamizole DILI.

	*n* = 45
Fibrosis	17 (37.8%)
Type of fibrosis^a^	
Collapsing fibrosis	3 (17.6%)
True fibrosis	14 (82.4%)
Fibrosis severity (stage according to Desmet)^a^	
Portal	6 (40.0%)
Portal‐septal	7 (46.7%)
Portal‐septal with changes of liver architecture	1 (6.7%)
Cirrhosis	1 (6.7%)
Inflammatory infiltrates	45 (100.0%)
Acute or chronic hepatitis^a^	
Acute hepatitis	4 (20.0%)
Chronic hepatitis	6 (30.0%)
Acute and chronic hepatitis	10 (50.0%)
Primary location of inflammatory infiltrates^a^	
Portal	21 (47.7%)
Portal and intralobular	18 (40.9%)
Intralobular	2 (4.5%)
In the necrotic areas	3 (6.8%)
Type of inflammatory infiltrates^a^	
Mixed	21 (51.2%)
Lympho‐ and plasmacellular	9 (22.0%)
Lymphocellular	5 (12.2%)
Lymphocytes and eosinophilic cells	4 (9.8%)
Lymphocytes and neutrophilic cells	2 (4.9%)
Interface hepatitis	20 (44.4%)
Plasma cells	22 (48.9%)
Lymphocytes	38 (84.4%)
Leucocytes	22 (48.9%)
Eosinophilic cells	31 (68.9%)
Cholestasis	19 (42.2%)
Necrosis	38 (84.4%)
Confluent multilobular necrosis	11 (28.2%)
Steatosis	9 (20.0%)
Severity of steatosis^a^	
Mild (< 30%)	7 (77.8%)
Moderate (30%–60%)	1 (11.1%)
Severe (> 60%)	0 (0.0%)
Ductular proliferation	13 (28.9%)
Ballooning of hepatocytes	7 (15.6%)
Lipofuscinosis	4 (8.9%)

*Note:* Variables are presented as numbers and percentages (*n* (%)).

^a^ Only cases for which the feature was present were included in the respective analysis; thus, data is presented as a percentage of the total number of patients for whom the respective feature was present. Cases not having a defined subtype were not included in the respective subgroup analysis.

### 3.6 Parameters Influencing Outcome

A relevant proportion of patients with metamizole DILI (*n* = 16, 26.2%) developed ALF, and 13.1% (*n* = 8) died or underwent LT. Patients with ALF showed higher AST, ALT, TBIL, INR, and MELD at onset and peak levels (Table S2). While remission rates were lower in patients with ALF, time to remission was comparable in those patients who went into remission (Table S2). In addition, the decline of AST and ALT until day 7 after DILI recognition on day 1 was significantly higher in patients with metamizole‐induced ALF (−22.7 × ULN vs. −2.1 × ULN, *p* < 0.001, and −18.1 × ULN vs. 2.4 × ULN, *p* = 0.002), while TBIL showed a significantly higher increase at day 3 (2.3 × ULN vs. 0.5 × ULN, *p* = 0.014; Table S2). Hy's law, as a conventional measure for prediction of ALF, was positive in 43 metamizole DILI patients. Out of those 43 patients, 16 developed ALF (37.2%), and all patients with ALF also were Hy's law positive, translating into a sensitivity of 100.0% and specificity of 37.2% for Hy's law predicting ALF in our metamizole DILI cohort (Table S2). Thus, due to low specificity, Hy's law was a suboptimal prediction marker for the development of ALF in our cohort. We therefore aimed to identify better prediction markers for adverse outcomes in our cohort of patients with metamizole DILI.

**FIGURE 4:**
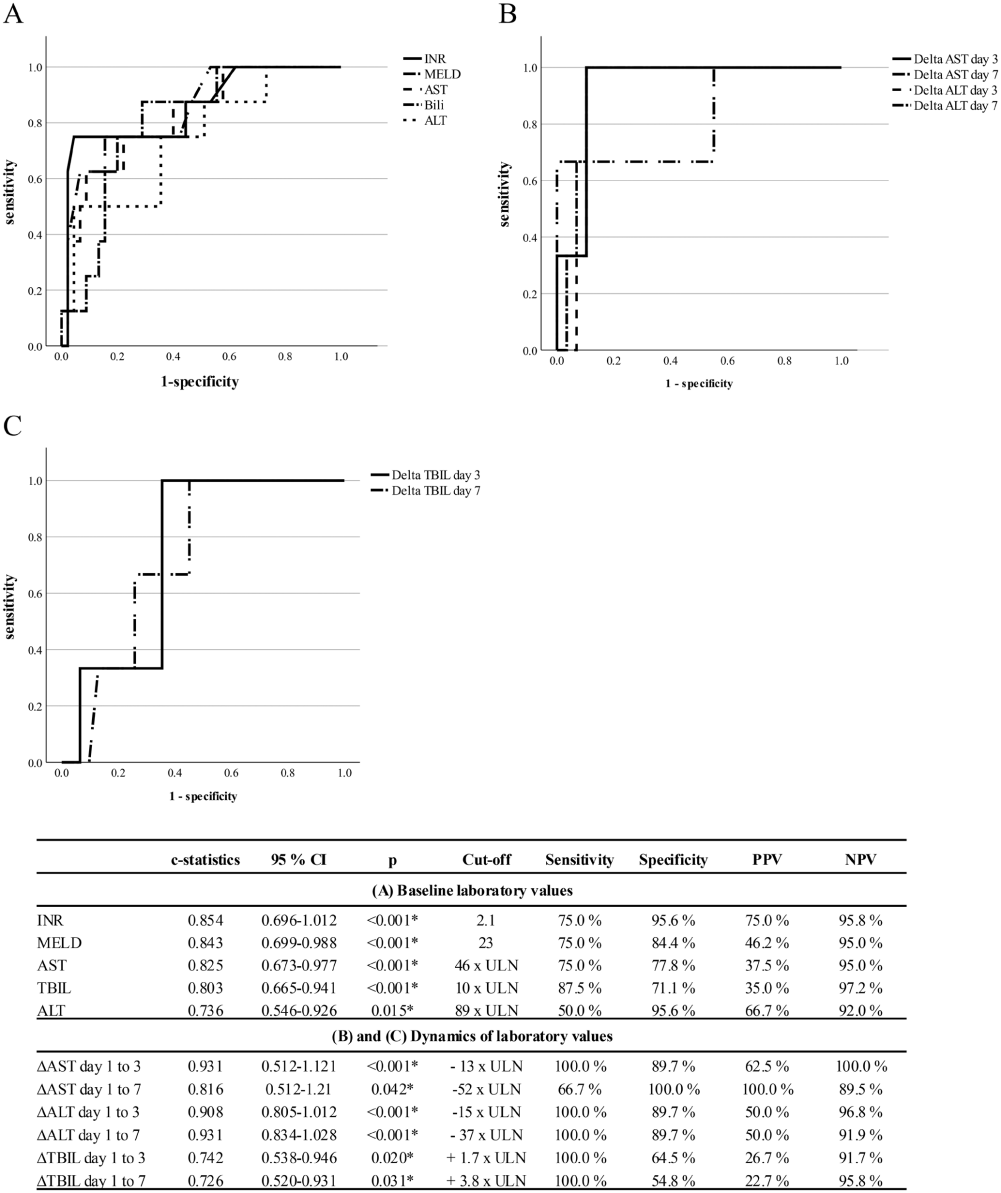
ROC analysis and predictive powers, sensitivity and specificity for laboratory parameters at the time of clinical presentation and for the time‐dependent dynamic changes of liver parameters regarding fatal adverse outcomes in metamizole DILI. Shown are the *c*‐statistics, sensitivity and specificity of (A) baseline parameters at the time of clinical presentation and scores, (B) the time‐dependent dynamic changes of AST, ALT and (C) the time‐dependent dynamic changes of TBIL regarding a fatal adverse outcome defined by death or liver transplantation. The cut‐offs were determined by ROC curve analysis and Youden's index. ALT, alanine aminotransferase; AST, aspartate aminotransferase; DILI, drug‐induced liver injury; INR, international normalised ratio; MELD, model for end‐stage liver disease; NPV, negative predictive value; PPV, positive predictive value; ROC, receiver of the operator characteristic; ROC, receiver operating characteristic; TBIL, total bilirubin. * statistical significance (*p* < = 0.05).

On univariate analysis, ALT, AST, TBIL, INR, and MELD at onset were significantly associated with fatal adverse outcomes (as defined by LT or death; Table S3). Age, sex, daily dosage of metamizole, or latency until onset of DILI did not have a significant effect on outcome; neither did corticosteroid treatment (Table S3). In the multivariate logistic regression analysis, only AST (odds ratio [OR]: 1.07, 95% confidence interval [CI]: 1.02–1.13, *p* = 0.009) and INR at onset (OR: 3.42, 95% CI: 1.47–7.93, *p* = 0.004, Table S4) remained significantly associated with a fatal adverse outcome. The *c*‐statistic for fatal adverse outcomes for INR, MELD, AST, TBIL, and ALT at onset were 0.85 (95% CI: 0.70–1.01, *p* < 0.001), 0.84 (95% CI: 0.70–0.99, *p* < 0.001), 0.83 (95% CI: 0.67–0.98, *p* < 0.001), 0.80 (95% CI: 0.67–0.94, *p* < 0.001), and 0.74 (95% CI: 0.55–0.92, *p* = 0.015; Figure 4). The optimal cut‐off for INR was determined at ≥ 2.1, at which a sensitivity of 75.0% and a specificity of 95.6% for a fatal adverse outcome were observed, while TBIL with a cut‐off of 10 × ULN showed the highest sensitivity (87.5%) at still reasonable specificity (71.1%; Figure 4). INR in particular showed an extraordinary discriminatory power with both high PPV (75.0%) and negative predictive values (NPV; 95.8%). In addition, while all of the baseline laboratory parameters showed high NPV above 90%, with 97.2% the highest NPV was observed for TBIL at a cut‐off of 10 × ULN. The time‐dependent dynamic changes of transaminases following DILI recognition performed even better in predicting fatal adverse outcomes: The *c*‐statistics for ΔAST from day 1 to 3, as well as ΔALT from day 1 to 3 and from day 1 to day 7, were all > 0.9 (*p* < 0.001; Figure 4). Sensitivity and specificity for a fatal adverse outcome were 100.0% and 89.7%, respectively, for a decline of AST of at least 13 × ULN by day 3 or a decline of ALT by at least 15 × ULN at day 3 and 37 × ULN at day 7 (Figure 4). With regards to PPV and NPV, ΔAST between day 1 and 7 at a cut‐off of −52 × ULN had the highest discriminatory power with a PPV of 100.0% and NPV of 89.5% (Figure 4).

## 4 Discussion

Following multiple reports on DILI due to metamizole, the hepatotoxic potential of metamizole is now well established [7, 8]. This is underlined by our finding that metamizole was associated with liver injury in 18.8% of 324 DILI patients included in our prospective study on ALI with a potential drug‐related cause. However, polypharmacy can hinder the identification of the causative agent. Metamizole as an analgesic and antipyretic agent is seldomly used alone, as it was demonstrated in a study on agranulocytosis induced by metamizole [21]. In fact, in our cohort only two of the 61 patients with metamizole DILI had been taking no medication other than metamizole, while the majority of patients from our cohort had been using co‐medications with a compatible timing to onset of liver injury. The concomitant medication was mainly comprised of NSAIDs and antimicrobial substances, two drug classes that have been described as most commonly associated with DILI [22]. Thus, due to those confounding co‐medications, metamizole might be underrecognized as a likely agent causing DILI. This can then lead to the erroneous discontinuation of innocent bystander medication and/or unintentional re‐exposure with metamizole. Alarmingly, re‐exposure to a medication that has previously caused DILI can lead to another DILI episode and in some cases also result in ALF and death [23]. In line with this, we found that nine patients from our cohort (14.8%) had an involuntary positive re‐challenge with metamizole leading to a secondary (and even tertiary) episode of DILI. We observed that these re‐exposures occurred when patients and their physicians had related DILI to the co‐medication rather than to metamizole, which led to the misperception of metamizole being a safe drug in the respective patients.

Overall, the phenotype of liver injury caused by metamizole which we observed in the current study, was comparable to previous data [7]. The majority of patients presented with a hepatocellular injury pattern with marked elevation of AST, ALT, and TBIL as well as high rates of positive ANA, low ALP values, as well as strong inflammatory activity and extensive necrosis on histological analysis. Interestingly, the clinical features of metamizole DILI and AIH patients were quite similar, while relevant differences were rather observed in comparison to patients with non‐metamizole DILI, in particular regarding the level of liver parameter elevation, which was generally lower in patients with non‐metamizole DILI. In addition to a high rate of ANA positivity, a relatively high proportion of metamizole DILI patients presented with AMA (25%). The prevalence of ANA is commonly associated with AIH, while AMA is mostly correlated with primary biliary cholangitis; however, as we have described earlier, relevant proportions of DILI patients can present with both ANA and AMA positivity [24]. Moreover, an unexpectedly high proportion of DILI patients had fibrosis (38%), which, however, was mostly mild to mild–moderate. Only one patient presented with severe fibrosis; however, this patient was transplanted due to severe DILI, and no other typical features for AIH were observed; most importantly, IgG was normal, and interface hepatitis was absent in the histological work‐up, which strongly indicates that DILI and not AIH was the cause of liver injury in this case. Due to the lack of specific features, differential diagnosis between DILI and AIH largely depends on clinical follow‐up [25, 26]. All of our cases were evaluated by RUCAM to complement thorough assessments of causality of liver injury performed by expert consensus. In addition, cases were followed up long‐term (median follow‐up of the metamizole cases: 29 [1–470] weeks) with no evidence of spontaneous recurrence of liver injury, which is one of the most accurate methods to distinguish DILI from AIH. Thus, despite relatively high rates of ANA positivity and fibrosis, we can provide a convincing differential diagnosis between DILI and AIH in our cohort.

Interestingly, we identified a characteristic phenotype in 43 of the 61 patients with metamizole DILI. This typical injury pattern included a hepatocellular type of injury with marked elevation of transaminases at the time of DILI onset (AST: 36.9 × ULN, ALT:43.6 × ULN), which generally peaked at the time of DILI recognition, and a delayed increase of bilirubin with a median peak 7 days after DILI onset. Patients with the metamizole DILI signature pattern could best be distinguished from controls by baseline ALT at a cut‐off of 29 × ULN or baseline TBIL at a cut‐off of 5.2 × ULN with a PPV of 87% and 83%, respectively. Moreover, we observed that the increase of TBIL in the first 3–7 days after DILI recognition was a crucial parameter when it comes to identifying metamizole DILI cases. As such, the time‐based change of TBIL from day 1 to day 3 was one of the only parameters that was significantly different in patients with the characteristic metamizole DILI when compared to each control group alone. Strikingly, an increase of TBIL above baseline values by at least 0.1 × ULN in the first 3 days after liver injury recognition could distinguish metamizole DILI from controls with a high discriminatory power (PPV: 97%, sensitivity 87%, specificity 86%).

Furthermore, patients with the characteristic metamizole DILI pattern showed a relatively long latency from the start of drug intake until the time of DILI recognition of 48 days. This is in line with the previous observations on metamizole DILI showing average latencies of 4–6 weeks and latencies as long as 35 weeks [7, 8]. In addition, 43% of patients with the characteristic metamizole DILI had discontinued the drug before DILI onset with a median time from drug discontinuation until the appearance of liver injury of 17 days. This time interval is longer than the latencies usually described for DILI, which are generally expected to be no longer than 15 days after drug withdrawal in case of earlier discontinuation [11]. However, longer latencies have also been observed for other agents, e.g., minocycline or nitrofurantoin, a fact that has been considered by the revised electronic causality assessment method (RECAM) recently proposed by Hayashi et al. [27].

Since 30% of all metamizole cases from our cohort only had a RUCAM below 6, meaning metamizole was an “unlikely” or “possible” cause for liver injury [18], we evaluated whether RUCAM had an influence on the severity of liver parameter elevation or the evolution of liver parameters. However, no significant differences could be observed regarding baseline or peak liver parameters nor the time‐dependent dynamic changes of serum transaminases or TBIL. Thus, our results indicate that metamizole DILI patients with RUCAM ≥ 6 or < 6 were comparable, arguing against the RUCAM score being a reliable discriminating tool in the assessment of metamizole DILI. It is well known that the RUCAM has major limitations, in particular in patients with polymedication or in severe cases [15, 28]. For instance, a higher score is given if the latency between drug initiation and DILI onset is between 5 and 90 days or ≤ 15 days after drug discontinuation. However, as described above, we observed longer latencies in some of our cases, in particular in patients that had discontinued metamizole before recognition of DILI. This might be due to a prolonged asymptomatic period until liver injury becomes apparent in metamizole DILI cases. In addition, simultaneous intake of other agents with DILI potential will lead to a lower RUCAM score for both metamizole and the concomitant medication. As we have pointed out, the majority of our patients were treated with a concomitant medication with compatible timing to the onset of liver injury, artificially leading to a lower RUCAM score for metamizole, which, however, does not reflect a lower probability of metamizole being the causative agent. Moreover, a relatively large proportion of patients presented with ALF, which, due to an increase of liver function tests after the onset of liver injury can lead to a lower score for the item “course of reaction,” however, once again, it does not lower the probability of metamizole causing DILI.

Apart from the severity of liver enzyme increase, the characteristic metamizole DILI signature included Hy's law positivity in the majority of cases, coagulopathy in 72% of the patients, and with 37% also a high rate of ALF. Strinkingly despite the high severity in the subgroup with the metamizole DILI signature pattern, recovery rates remained high at 74%, which were also comparable to the recovery rates observed in larger registry studies on DILI cases in general [22, 29]. In addition, despite the higher severity and the more prominent increases in liver enzymes, no significant differences regarding the rates of recovery were observed between patients with the metamizole DILI signature pattern and control cases. Our findings therefore indicate that the characteristic metamizole DILI signature pattern is characterised by high severity at the time of DILI onset but not by higher mortality rates or lower remission rates.

With high peak serum transaminase and rapid reversal of these changes after drug discontinuation, the biochemical profile of metamizole DILI, which was identified by us, has some similarities with acetaminophen overdose [11]. Nevertheless, the timing to onset of liver injury after drug exposure differs significantly between those agents. In addition, while similarly to acetaminophen overdose, necrosis was highly present in our patients with metamizole DILI, histopathological findings also showed substantial differences when compared to those commonly observed in acetaminophen‐induced liver injury [11].

The characteristic metamizole DILI signature pattern can potentially help clinicians in the future when confronted with a patient with suspected DILI and intake of metamizole among other agents. We propose that the intake of metamizole together with a marked elevation of transaminases at the time of liver injury detection, a secondary peak of bilirubin, and a reported latency from the start of the drug intake of approximately 7–8 weeks should prompt the diagnosis of a metamizole‐induced liver injury. In this regard, our data suggests that patients with baseline ALT of ≥ 29 × ULN or TBIL of ≥ 5.2 × ULN, as well as an increase of TBIL of ≥ 0.1 × ULN during the 3 days after onset of liver injury, are more prone to suffering from metamizole DILI than an alternative cause of liver injury or DILI caused by alternative agents. While these cut‐offs were established for the metamizole DILI subgroup with the typical injury pattern, there is the possibility that the distinct subtypes of metamizole DILI presented here might merely reflect that liver injury became apparent at a different time point in the evolution of DILI. For a further evaluation and validation of the signature pattern identified by us, the clinical features of metamizole DILI should be evaluated in prospective DILI cohorts and then be compared to larger control groups in the future.

Since DILI is one of the leading causes of ALF [30], and metamizole is a relevant cause of DILI as demonstrated by our data, prediction of a fatal adverse outcome defined by death or LT in metamizole DILI has major clinical implications. We therefore aimed to further identify parameters or scores that can help to predict which patients with metamizole DILI are at high risk for a fatal adverse outcome even before the typical metamizole pattern becomes apparent. In this regard, Hy's law had a high sensitivity of 100% but a low specificity of only 37% for developing ALF in our cohort, which is comparable to previous data [31, 32]. On multivariate analysis, the only baseline parameters significantly associated with fatal adverse outcomes as defined by death or LT were baseline INR and AST values. INR in particular showed the highest discriminatory power, with a NPV of 96% and a PPV of 75% at a cut‐off of ≥ 2.1. Strinkingly when the time‐dependent dynamic changes of liver parameters after DILI recognition were evaluated, the predictive power regarding a fatal adverse outcome could be drastically improved: A fatal adverse outcome could best be predicted within 3 days after clinical presentation if a decline of AST of at least 13 × ULN or ALT of at least 15 × ULN was observed (sensitivity 100%, specificity 90%). Moreover, a decline of AST by ≥ 52 × ULN 7 days after DILI recognition was highly associated with a fatal adverse outcome (PPV 100%, NPV 90%). Thus, in the case of a marked coagulopathy with an INR of 2.1 or higher at the time of DILI recognition or a decline of AST of at least 13 × ULN within the first 3 days or 52 × ULN during the first week after DILI onset, patients are at a particular high risk of a fatal adverse outcome and should rapidly be transferred to a transplant centre since the risk for death or LT is high.

Our study has limitations. The control groups, for instance, to which the patients with the characteristic metamizole DILI pattern were compared to, were limited in sample size and were rather heterogeneous. In addition, due to the lack of a general gold standard for DILI diagnosis and causality assessment in patients with multiple drug intake, one might argue that causality assessment and, in particular, differentiation from AIH cannot be conducted in a standardised manner. While the latter limitation is inherently unavoidable in clinical studies on DILI, the strengths of our study are the large sample size of patients with metamizole DILI, the thorough clinical work‐up, as well as the long‐term follow‐up enabling a detailed causality assessment. In addition, a relatively large proportion of our patients presented with recurrence of liver injury upon re‐exposure towards metamizole, which is a strong indicator of this drug being the underlying cause of liver injury [13, 33]. Moreover, the comparison to patients with non‐metamizole DILI or alternative causes for liver injury despite metamizole intake provides the possibility to diagnose or exclude metamizole as the causative agent based on the clinical picture. Our findings should stimulate further prospective studies evaluating the accuracy and efficacy of the proposed metamizole signature and outcome markers.

In summary, we have identified a characteristic metamizole DILI phenotype comprised of high transaminases at the time of DILI onset, a secondary increase of TBIL, and significant proportions developing ALF but still high remission rates. Indicators for a fatal adverse outcome are high AST and especially INR at the time of DILI recognition, as well as a drastic decline of AST in the first 3–7 days after hospitalisation, which should lead to rapid liver transplant evaluation in order to avoid death from ALF.

## Author Contributions

Conceptualization: S.W., F.E. and A.L.G.; methodology: S.W. and F.E.; software: S.W. and F.E.; investigation: S.W., F.E., J.A., D.S., N.D., J.N. and C.M.L.; formal analysis: S.W., F.E. and J.N.; validation: S.W., F.E. and A.L.G.; resources: S.W., J.A., C.M.L. and A.L.G.; data curation: S.W. and F.E.; writing – original draft: S.W.; writing – review and editing: S.W., F.E., J.N., C.M.L. and A.L.G.; visualisation: S.W., F.E. and J.N.; supervision: S.W. and A.L.G.; project administration: S.W. and A.L.G.; funding acquisition: S.W. and A.L.G. All authors approved the final version of the manuscript.

## Ethics Statement

The study protocol conforms to the ethical guidelines of the Declaration of Helsinki and was approved by the ethics committee of the Faculty of Medicine, LMU Munich (Project Number 55‐13).

## Consent

Written informed consent was obtained from each participant.

## Conflicts of Interest

The authors declare no conflicts of interest.

## Supporting information


Table S1

Table S2

Table S3

Table S4

## Data Availability

All data generated or analysed during this study are included in this article. Further enquiries can be directed to the corresponding author.
