# Supplementary material for: Drug‐Induced Liver Injury Caused by Metamizole: Identification of a Characteristic Injury Pattern
Source: Liver Int. 2025 Feb 6;45(3):e70012. doi: 10.1111/liv.70012 (PMC11801327; doi:10.1111/liv.70012)
Supplement: Supplementary file 1 — Table S1 [file LIV-45-0-s003.docx]

**Suppl. Table 1 Comparison of laboratory evolution in patients with metamizole DILI according to RUCAM scoring**

|  | **RUCAM < 5**  **n=18** | **RUCAM ≥ 6**  **n=43** | **p** |
| --- | --- | --- | --- |
| **At the time of DILI recognition** | | | |
| **AST (xULN)** | 33.9 (1.1-85.9) | 26.7 (2.7-115.3) | 0.740 |
| **ALT (xULN)** | 40.4 (5.8-98.9) | 33.4 (3.8-138.5) | 0.937 |
| **ALP (xULN)** | 1.5 (0.6-3.7) | 1.9 (0.8-13.7) | 0.208 |
| **TBIL (xULN)** | 10.0 (0.2-24.3) | 6.0 (0.4-22.1) | 0.164 |
| **INR** | 1.8 (0.9-6.1) | 1.3 (0.8-3.0) | **0.023*** |
| **MELD** | 21 (6-35) | 18 (6-30) | 0.104 |
| **R-ratio ^†^** | 23.3 (3.5-83.1) | 19.3 (0.4-63.0) | 0.849 |
| **Peak values** | | | |
| **AST (xULN)** | 39.3 (1.7-85.9) | 28.8 (3-1115.3) | 0.689 |
| **ALT (xULN)** | 45.5 (6.9-101.0) | 38.0 (5.9-145.5) | 0.912 |
| **ALP (xULN)** | 1.6 (1.0-5.7) | 2.2 (1.0-15.4) | 0.229 |
| **TBIL (xULN)** | 18.4 (0.5-32.3) | 13.5 (0.7-34.5) | 0.438 |
| **INR** | 1.9 (0.9-8.0) | 1.5 (0.9-7.2) | **0.032*** |
| **MELD** | 25 (6-40) | 20 (6-40) | 0.097 |
| **R-ratio ^†, ‡^** | 25.0 (4.1-91.5) | 23.8 (0.4-116.5) | 0.693 |
| **Time-dependent dynamic changes** **of liver parameters** ^§^ | | | |
| Δ**AST day 1 to day 3 (xULN)** | -5.3 (-36.3-2.8) | -1.7 (-21.6-13.8) | 0.168 |
| Δ**AST day 1 to day 7 (xULN)** | -19.1 (-76.4-25.7) | -10.8 (-63.6-20.4) | 0.123 |
| Δ**ALT day 1 to day 3 (xULN)** | -8.9 (-33.1-1.8) | -5.9 (-59-15.5) | 0.446 |
| Δ**ALT day 1 to day 7 (xULN)** | -17.6 (-77.4-22.4) | -5.0 (-79.2-23.1) | 0.060 |
| Δ**TBIL day 1 to day 3 (xULN)** | 2.7 (-0.08-6.20) | 0.7 (-5.2-12.4) | 0.064 |
| Δ**TBIL day 1 to day 7 (xULN)** | 4.2 (-0.3-12.0) | 2.7 (-8.0-18.4) | 0.317 |

Variables are presented as median (range). ^†^ The R-ratio is defined as (ALT/ULN)/(ALP/ULN), with R ≥5 defining a hepatocellular, R ≤2 a cholestatic and 2<R<5 a mixed type of injury. ^‡^ Peak R-ratio was calculated on the date of peak ALT for hepatocellular and mixed type of injury and on the date of peak ALP for cholestatic type of injury. ^§^ Under this section, the time-based changes of the respective liver parameters from day 1 to day 3 or day 7 expressed as fold ULN are shown. * indicates a statistical significance (p≤0.05).

Abbreviations: ALP: Alkaline phosphatase; ALT: Alanine aminotransferase; AST: Aspartate aminotransferase; DILI: Drug-induced liver injury; INR: International normalized ratio; MELD: Model for end-stage liver disease; RUCAM: Roussel Uclaf Causality Assessment Method; TBIL: Total bilirubin; ULN: Upper limit of normal.
