# Supplementary material for: Drug‐Induced Liver Injury Caused by Metamizole: Identification of a Characteristic Injury Pattern
Source: Liver Int. 2025 Feb 6;45(3):e70012. doi: 10.1111/liv.70012 (PMC11801327; doi:10.1111/liv.70012)
Supplement: Supplementary file 2 — Table S2 [file LIV-45-0-s001.docx]

**Suppl. Table 2 Comparison of clinical and laboratory parameters in patients with metamizole-induced ALF vs. metamizole DILI without ALF**

|  | **ALF**  **n=16** | **no ALF**  **n=45** | **p** |
| --- | --- | --- | --- |
| **Clinical parameters** | | | |
| **Age (years)** | 44 (26-64) | 44 (19-84) | 0.909 |
| **Body mass index (kg/m2)** | 23.7 (18.1-31.0) | 24.1 (18.9-37.9) | 0.609 |
| **Female** | 9 (56.3 %) | 33 (73.3 %) | 0.205 |
| **Average daily dosage metamizole (mg)** | 1000 (200-2000) | 1000 (71-4000) | 0.798 |
| **Latency from start of drug intake until onset of DILI (days)** | 77 (24-674) | 34 (2-102) | **0.006*** |
| **Latency from last day of intake until onset of DILI (days) ^†^** | 22 (1-106) | 10 (1-52) | 0.286 |
| **Hy’s law positivity** | 16 (100.0%) | 27 (60.0 %) | **0.003*** |
| **Outcome** | | | |
| **Liver transplantation** | 6 (37.5 %) | 0 (0.0 %) | **<0.001*** |
| **Death** | 1 (6.3 %) | 1 (2.2 %) |  |
| **Remission** | 9 (56.3 %) | 38 (84.4 %) |  |
| **Chronicity** | 0 (0.0 %) | 1 (2.2 %) |  |
| **Time to remission (weeks)** | 11 (3-168) | 9 (3-81) | 0.416 |
| **Laboratory parameters at the time of DILI recognition** | | | |
| **AST (xULN)** | 48.5 (22.5-115.3) | 21.7 (1.1-68.5) | **<0.001*** |
| **ALT (xULN)** | 50.1 (14.9-138.5) | 31.6 (3.8-105.5) | **0.004*** |
| **ALP (xULN)** | 2.2 (1.0-5.2) | 1.6 (0.6-13.7) | 0.184 |
| **TBIL (xULN)** | 13.2 (6.0-24.3) | 4.5 (0.2-20.0) | **<0.001*** |
| **INR** | 2.0 (1.3-5.3) | 1.2 (0.8-6.1) | **<0.001*** |
| **MELD** | 24 (19-31) | 15 (6-35) | **<0.001*** |
| **R-ratio ^†^** | 24.0 (4.4-83.1) | 19.3 (0.4-65.4) | 0.195 |
| **Laboratory parameters at peak values** | | | |
| **AST (xULN)** | 53.1 (22.5-115.3) | 26.0 (1.7-95.3) | **<0.001*** |
| **ALT (xULN)** | 53.0 (14.9-145.5) | 35.2 (5.9-105.9) | **0.012*** |
| **ALP (xULN)** | 2.6 (1.3-5.7) | 1.9 (1.0-15.4) | 0.060 |
| **TBIL (xULN)** | 22.1 (13.5-34.5) | 6.9 (0.5-29.1) | **<0.001*** |
| **INR** | 2.4 (1.5-8.0) | 1.3 (0.9-6.1) | **<0.001*** |
| **MELD** | 27 (19-40) | 16 (6-35) | **<0.001*** |
| **R-ratio ^‡,^** ^§^ | 24.0 (4.4-91.5) | 23.8 (0.4-116.1) | 0.491 |
| **Time-dependent dynamic changes** **of liver parameters ^¶^** | | | |
| Δ**AST day 1 to day 3 (xULN)** | -7.9 (-36.3-13.8) | -2.0 (-21.0-9.3) | 0.406 |
| Δ**AST day 1 to day 7 (xULN)** | -22.7 (-76.4-(-8.6)) | -2.1 (-33.0-25.7) | **<0.001*** |
| Δ**ALT day 1 to day 3 (xULN)** | -8.3 (-38.5-15.5) | -5.3 (-59.0-8.3) | 0.288 |
| Δ**ALT day 1 to day 7 (xULN)** | -18.1 (-79.2-2.5) | -2.4 (-58.6-23.1) | **0.002*** |
| Δ**TBIL day 1 to day 3 (xULN)** | 2.3 (-5.2-12.4) | 0.5 (-1.5-6.8) | **0.014*** |
| Δ**TBIL day 1 to day 7 (xULN)** | 7.8 (-8.0-17.3) | 2.3 (-3.4-18.4) | 0.066 |

Categorical variables are presented as number and percentage (n (%)). Continuous variables are presented as median (range). ^†^ In case metamizole was discontinued before onset of liver injury. ^‡^ The R-ratio is defined as (ALT/ULN)/(ALP/ULN), with R ≥5 defining a hepatocellular, R ≤2 a cholestatic and 2<R<5 a mixed type of injury. ^§^ Peak R-ratio was calculated on the date of peak ALT for hepatocellular and mixed type of injury and on the date of peak ALP for cholestatic type of injury. **^¶^** Under this section, the time-based changes of the respective liver parameters from day 1 to day 3 or day 7 expressed as fold ULN are shown. * indicates a statistical significance (p≤0.05).

Abbreviations: ALF: Acute liver failure; ALP: Alkaline phosphatase; ALT: Alanine aminotransferase; AST: Aspartate aminotransferase; DILI: Drug-induced liver injury; IgG: Immunoglobulin G; INR: International normalized ratio; MELD: Model for end-stage liver disease; TBIL: Total bilirubin; ULN: Upper limit of normal.
