# Supplementary material for: Drug‐Induced Liver Injury Caused by Metamizole: Identification of a Characteristic Injury Pattern
Source: Liver Int. 2025 Feb 6;45(3):e70012. doi: 10.1111/liv.70012 (PMC11801327; doi:10.1111/liv.70012)
Supplement: Supplementary file 3 — Table S3 [file LIV-45-0-s002.docx]

**Suppl. Table 3 Univariate logistic regression analysis for adverse outcome in patients with metamizole DILI**

|  | **OR** | **95 % CI** | **p** |
| --- | --- | --- | --- |
| **Female sex** | 1.043 | 0.838-1.298 | 0.722 |
| **Age at diagnosis** | 0.997 | 0.946-1.051 | 0.918 |
| **Daily dosage metamizole** | 0.999 | 0.997-1.001 | 0.191 |
| **Latency from start of drug intake** | 1.001 | 0.994-1.008 | 0.861 |
| **R ratio at onset ^†^** | 1.030 | 0.991-1.071 | 0.139 |
| **AST at onset** | 0.749 | 0.587-0.957 | **0.021*** |
| **ALT at onset** | 1.029 | 1.004-1.053 | **0.022*** |
| **ALP at onset** | 0.957 | 0.497-1.843 | 0.896 |
| **TBIL at onset** | 1.155 | 1.036-1.289 | **0.010*** |
| **INR at onset** | 1.197 | 1.040-1.377 | **0.012*** |
| **MELD at onset** | 1.264 | 1.057-1.511 | **0.010*** |
| **Characteristic metamizole DILI pattern** | 1.250 | 1.071-1.459 | 0.053 |
| **Hy’s law positive** | 1.242 | 1.069-1.445 | 0.065 |
| **ANA positivity** | 1.004 | 0.783-1.287 | 0.975 |
| **AMA positivity** | 1.000 | 0.765-1.307) | 1.000 |
| **IgG** | 1.061 | 0.898-1.253 | 0.485 |
| **Corticosteroid treatment** | 0.697 | 0.194-2.506) | 0.579 |

Shown are the odds ratios (OR) and the 95% confidence intervals (CI) for baseline and dynamic laboratory parameters with regards to adverse outcome in univariate analysis. Adverse outcome was defined as death or liver transplantation. ^†^ The R-ratio is defined as (ALT/ULN)/(ALP/ULN), with R≥5 defining a hepatocellular, R≤2 a cholestatic and 2<R<5 a mixed type of injury. * indicates a statistical significance (p≤0.05).

Abbreviations: ALP: Alkaline phosphatase; ALT: Alanine aminotransferase; AMA: Anti-mitochondrial antibodies; ANA: Antinuclear antibodies; AST: Aspartate aminotransferase; IgG: Immunoglobulin G; INR: International normalized ratio; MELD: Model for end-stage liver disease; TBIL: Total bilirubin.
