# Supplementary material for: Drug‐Induced Liver Injury Caused by Metamizole: Identification of a Characteristic Injury Pattern
Source: Liver Int. 2025 Feb 6;45(3):e70012. doi: 10.1111/liv.70012 (PMC11801327; doi:10.1111/liv.70012)
Supplement: Supplementary file 4 — Table S4 [file LIV-45-0-s004.docx]

**Suppl. Table 4 Multivariate logistic regression regarding fatal adverse outcomes in metamizole DILI**

|  | **OR** | **95 % CI** | **p** |
| --- | --- | --- | --- |
| **AST** | 1.071 | 1.017-1.127 | **0.009*** |
| **INR** | 3.415 | 1.470-7.932 | **0.004*** |

Shown are the results of the multivariate logistic regression analysis regarding fatal adverse outcomes defined by death or liver transplantation. Variables considered in the multivariate analysis were baseline parameters with p<0.100 in univariate analysis. Variables excluded by backward logistic regression were ALT, TBIL, MELD, Hy’s law positivity and the characteristic metamizole DILI pattern.

Abbreviations: ALT: Alanine aminotransferase; AST: Aspartate aminotransferase; CI: Confidence interval; DILI: Drug-induced liver injury; INR: International normalized ratio; MELD: Model for end-stage liver disease; OR: Odds ratio; TBIL: Total bilirubin.
